# CCL4在肿瘤微环境中影响免疫逃逸的研究进展

**DOI:** 10.3779/j.issn.1009-3419.2024.106.23

**Published:** 2024-08-20

**Authors:** Ran CHEN, Xinyue YANG, Qian LIU, Shucai ZHANG, Li MA

**Affiliations:** ^1^441000 襄阳，湖北医药学院附属襄阳市第一人民医院肿瘤科（陈然，刘倩）; ^1^Department of Oncology, Xiangyang No.1 People's Hospital, Hubei University of Medicine, Xiangyang 441000, China; ^2^101149 北京，首都医科大学附属北京胸科医院北京市结核病胸部肿瘤研究所肿瘤内科（陈然，张树才，马丽）; ^2^Department of Medical Oncology, Beijing Chest Hospital, Capital Medical University, Beijing Tuberculosis and Thoracic Tumor Research Institute, Beijing 101149, China; ^3^441000 襄阳，武汉科技大学医学院襄阳市第一人民医院研究生联合培养基地肿瘤科（杨新月）; ^3^Department of Oncology, Postgraduate Union Training Base of Xiangyang No.1 People's Hospital, School of Medicine, Wuhan University of Science and Technology, Xiangyang 441000, China

**Keywords:** CCL4, 免疫治疗, 肿瘤微环境, 免疫应答, 免疫逃逸, CCL4, Immunotherapy, Tumor microenvironment, Immune response, Immune escape

## Abstract

免疫治疗已成为当下恶性肿瘤治疗的基石。然而，不同患者对免疫治疗的反应具有较大异质性，并非都能从中获益。当下亟需寻找能高效预测免疫治疗疗效的生物标志物。C-C趋化因子配体4（C-C chemokine ligand 4, CCL4）是一种细胞因子，隶属于炎症性CCL亚家族，主要由免疫细胞、肿瘤细胞分泌，在正常组织中不表达或者弱表达，在多种恶性肿瘤组织中异常高表达。CCL4与其受体C-C趋化因子受体5（C-C chemokine receptor type 5, CCR5）结合后可招募并介导免疫细胞迁移，破坏肿瘤微环境（tumor microenvironment, TME）的稳定性，参与致癌并促进肿瘤发展。在肿瘤免疫微环境中，CCL4可介导并募集单核细胞、巨噬细胞、自然杀伤（natural killer, NK）细胞、T细胞等关键免疫细胞定向迁移，成为影响免疫治疗疗效的潜在重要元素，而具有特定的价值。本文就CCL4在TME中影响免疫逃逸的研究进展进行综述，以期为基础研究及临床诊疗提供线索和参考。

## Abstract (en)

Immunotherapy has become the cornerstone of current malignant tumor treatment. However, the response of different patients to immunotherapy is highly heterogeneous, and not all patients can benefit from it. There is an urgent need to find biomarkers that can effectively predict the efficacy of immunotherapy. C-C chemokine ligand 4 (CCL4) is a cytokine, belonging to the inflammatory CCL subfamily. It is mainly secreted by immune cells and tumor cells and shows low or no expression in normal tissues but abnormally high expression in various malignant tumor tissues. After binding to CCL4 and its receptor C-C chemokine receptor type 5 (CCR5), it can recruit and mediate immune cell migration, destroy the stability of the tumor microenvironment (TME), participate in carcinogenesis and promote the development of tumors. In the tumor immune microenvironment, CCL4 can mediate and recruit the directed migration of key immune cells such as monocytes, macrophages, natural killer (NK) cells, and T cells, which makes it a potentially important element affecting the efficacy of immunotherapy and has specific value. This paper reviews the research progresses of CCL4's effects on immune escape in TME, in order to provide clues and references for basic research and clinical diagnosis and treatment.

恶性肿瘤的发生发展、浸润转移、复发生长等病理生理过程极其复杂，不仅与患者的遗传易感性相关，还与机体的免疫状态、肿瘤微环境（tumor microenvironment, TME）相关，是机体内环境免疫系统与肿瘤之间清除、平衡和逃逸的博弈。TME为肿瘤细胞所处的内环境，其中不仅包括肿瘤细胞，还包括与其关系密切的免疫细胞、基质细胞等关键成分^[1]^。近年来，以免疫检查点抑制剂（immune checkpoint inhibitors, ICIs）为代表的免疫治疗大大改善了多种恶性肿瘤的治疗疗效和预后，然而疗效并不确切。TME中的众多因素影响了免疫治疗的疗效，诸如其中肿瘤细胞可通过分泌细胞因子、趋化因子、间质代谢产物等生化分子来调控肿瘤与其他细胞之间的免疫应答及信号转导，从而影响免疫细胞的抗肿瘤活性^[2]^。

免疫逃逸是指肿瘤细胞通过各种机制避免免疫系统的识别和攻击而继续生长的现象，是恶性肿瘤对免疫治疗耐药或抵抗的最主要原因，也是肿瘤能够生存和发展的关键^[3,4]^。导致肿瘤发生免疫逃逸的因素众多且各异，如肿瘤特异性识别为自身抗原、肿瘤细胞的低免疫原性、肿瘤诱导的豁免区、肿瘤表面抗原的调节、肿瘤诱导产生的免疫抑制等。这些主要的因素和机制中，肿瘤诱导产生的免疫抑制是当下研究的最为广泛和深刻的机制之一，其主要包括两种形式：（1）诱导免疫抑制分子，即免疫检查点分子的表达。大量的临床及基础研究^[5⇓-7]^表明，TME中肿瘤细胞和免疫细胞上免疫检查点分子的上调和持续表达是肿瘤发生免疫逃逸的重要机制，如程序性细胞死亡蛋白配体1（programmed cell death ligand 1, PD-L1）、细胞毒性T淋巴细胞相关蛋白4（cytotoxic T lymphocyte-associated antigen-4, CTLA-4）、T淋巴细胞免疫球蛋白黏蛋白3（T cell immunoglobulin domain and mucin domain-3, TIM-3）、淋巴细胞活化基因3（lymphocyte activation gene-3, LAG-3）等。免疫检查点分子与其特异性受体结合后，可有效抑制效应T细胞的活性，使得效应T细胞丧失活性，无法识别并杀灭癌细胞而产生免疫逃逸。通过ICIs阻断免疫检查点分子受体与配体的结合来解除免疫抑制，恢复效应免疫细胞的活性最终可达到抗肿瘤的目的^[8]^。目前程序性细胞死亡受体1（programmed cell death 1, PD-1）相关的ICIs如帕博丽珠单抗等已经在非小细胞肺癌（non-small cell lung cancer, NSCLC）^[9]^、胃癌^[10]^、黑色素瘤^[11]^等多瘤种中获得了巨大的成功。（2）通过诱导TME中肿瘤细胞、免疫细胞或基质细胞分泌产生细胞因子、趋化因子及代谢产物等生化分子及炎症免疫介质来调控细胞间的免疫应答、信号传导等关键活动及免疫细胞的浸润水平^[1]^，从而影响并改变TME的稳定性，最终达到抗肿瘤效应。

本综述所讨论的内容将侧重于上述肿瘤诱导免疫逃逸机制的第二种形式，重点关注TME中的细胞因子、趋化因子等免疫及炎症介质在抗肿瘤免疫应答和免疫逃逸中的特点和作用，来阐述其在抗肿瘤免疫应答中的重要价值。

## 1 C-C趋化因子配体4（C-C chemokine ligand 4, CCL4）概述

以趋化因子为主的细胞因子亚类是多种类型细胞病理生理活动中分泌的强而有力的调节剂，尤其在抗肿瘤免疫系统中，对恶性肿瘤的发生发展产生着重大的影响。趋化因子是一种小分子、分泌性且与细胞因子结构密切相关的细胞因子重要亚型，在炎症和免疫调节过程中起着关键作用，也是癌症相关炎症的关键介质，同时也是致癌途径中的靶点^[12]^。趋化因子最初被认为只是在决定肿瘤基质组成方面起着重要作用，后来发现他们能够直接影响癌症细胞的增殖和转移^[13,14]^，可能在抗肿瘤免疫治疗中具有预测免疫治疗疗效的潜在价值。

CCL4又被称作巨噬细胞炎症蛋白1β（macrophage inflammatory protein-1β, MIP-1β），属于炎症性CCL亚家族^[15]^。趋化因子被定义为控制免疫细胞迁移和定位的趋化型细胞因子，属于细胞因子家族，是一种与G蛋白偶联受体结合并诱导调节免疫细胞运输的细胞内级联的小分子蛋白，包括40多种不同的分子^[16]^。趋化因子在氨基酸水平上显示出较低的总体相似性。它在结构上与保守的N-末端半胱氨酸基序同源，根据其结构差异，可分为C-X-、C-、C-C-和C-X_3_-C-大致4类，其中X表示所存在的氨基酸。趋化因子受体根据相应的趋化因子命名为CXCR-、CCR-、CR-和CX_3_CR-^[17]^。人类CCL4基因位于染色体17q12，CCL4蛋白是在从其前体形式中去除含有23个氨基酸的信号肽以后，由69个氨基酸组成的成熟形式分泌而成^[18]^。

## 2 CCL4及其受体在TME中的作用和特点

### 2.1 CCL4的来源及其在人类肿瘤中的表达

最初的研究^[19]^提示趋化因子及其受体仅由免疫细胞表达，诸如粒细胞、单核细胞、巨噬细胞和淋巴细胞等。随后的研究^[20]^也表明，一些趋化因子受体也由非免疫细胞表达，包括内皮细胞、上皮细胞、成纤维细胞和癌症细胞等。除了以上倾向于认为趋化因子主要由免疫细胞和少量非免疫细胞分泌表达这些观点之外，后来大量研究^[21,22]^结果也证实肿瘤细胞是趋化因子的主要来源。有学者^[21]^研究发现结肠癌肿瘤细胞是TME中CCL4的最主要来源。支持这一观点的依据是β-catenin激活突变可以增强结肠癌细胞系中CCL4基因的表达，这是结肠癌中常见的基因突变^[23]^。然而CCL4在结肠癌发生发展中的作用却存在争议。据报道^[24]^高血清CCL4水平与改善结肠癌患者的无病生存期以及肿瘤周围CD68^+^巨噬细胞密度增加相关。直肠癌肿瘤细胞衍生的CCL4可以通过诱导原癌巨噬细胞浸润以及其他趋化因子如CCL2和CCL3^[21]^或通过招募调节性T细胞^[22]^（regulatory T cells, Tregs）来抑制肿瘤免疫，从而促进人类结肠癌的进展。

CCL4在大部分正常组织中不表达或者弱表达，但在肺腺癌、结直肠癌、乳腺癌等组织中异常高表达^[25,26]^，往往与不良预后相关^[27]^。在肺腺癌中的研究^[27]^表明，高血清CCL4浓度可预测肺腺癌的不良生存率。与肺鳞癌组织相比，CCL2和CCL4在肺腺癌组织中的表达水平增高，CCL4水平较高的肺腺癌患者总生存期（overall survival, OS）有缩短趋势^[27]^。也有研究^[28]^发现，在人类的口腔鳞状细胞癌（human oral squamous cell carcinoma, OSCC）组织中也检测到了CCL4的表达。CCL4可以在体外诱导肿瘤细胞表达血管内皮生长因子（vascular endothelial growth factor, VEGF），VEGF在血管淋巴管的形成中起着关键的作用。当把OSCC细胞系移植到裸鼠模型中的时候，发现CCL4的表达抑制了VEGF的表达和淋巴管的形成^[29]^，从而证明了CCL4不仅可以通过体外诱导由肿瘤细胞产生，还证明了CCL4在影响肿瘤淋巴管形成过程中所存在的意义和价值。

关于CCL4的特异性受体鉴定的相关研究中，Samson等^[30]^基于既往所观察到CCL3、CCL4这些趋化因子在其生理浓度下所具有的活性的显著差异，最终将C-C趋化因子受体5（C-C chemokine receptor type 5, CCR5）鉴定为CCL3、CCL4、CCL5的特异性受体。CCR5是一种七跨膜G蛋白偶联体，相应配体结合不同的信号转导级联，参与导致G蛋白激活和信号转导级联的激活^[31]^。CCR5主要由树突状细胞、T淋巴细胞、巨噬细胞、嗜酸性粒细胞及小胶质细胞等所表达^[32]^，也在多种肿瘤细胞中过表达，如霍奇金淋巴瘤^[33]^、乳腺癌^[34]^、胃腺癌^[35]^、胰腺癌^[36]^、黑色素瘤^[37]^等。

### 2.2 CCL4影响肿瘤发生发展的机制

CCL4与其特异性受体CCR5结合后协同相关的趋化因子如CCL3、CCL5等，对TME中各种类型的免疫细胞或非免疫细胞产生不同的趋化作用。首先，它可以通过招募Tregs及肿瘤相关巨噬细胞（tumor-associated macrophages, TAMs）作用于TME中其他驻留细胞，如成纤维细胞、内皮细胞等，破坏肿瘤免疫微环境的稳定性及抗肿瘤免疫功能，参与致癌^[38]^并促肿瘤生长^[39]^。其次，CCL4是一种在免疫应答、信号通路传导、炎症和肿瘤发生发展中发挥着关键作用的趋化剂^[40]^，如CCL4可以通过影响前列腺癌依赖的STAT3信号通路来促进肿瘤进展。越来越多的证据^[28,41]^表明了CCL4控制多种肿瘤的相关功能，如增殖、侵袭、转移和血管生成，且在不同的肿瘤类型中起着关键作用^[42,43]^。另外，包括CCL4在内的趋化因子是TME中负责免疫细胞的主要调节因子，在TME中调节免疫细胞聚集趋向的趋化因子与趋化因子受体结合后可协调改变TME中的免疫细胞构象^[44]^，从而对免疫细胞及肿瘤细胞产生直接和间接作用。研究^[45]^发现TME中的耗竭T细胞（T cell exhaustion, Tex）分泌的CCL4/CCL5等趋化因子能够积极招募单核细胞并促进其分化成抗原呈递态的TAMs。TAMs利用抗原呈递，与CD8^+ ^T细胞之间形成了一种独特且持久的“突触”作用。这种抗原特异性的相互作用非但不能激活T细胞活性，反而会引诱T细胞发生耗竭，尤其在肿瘤内部区域，TAMs借助乏氧环境，更是加速了T细胞耗竭的进程（图1），特别是在肺癌的微环境中TAMs可以通过极化为具有免疫抑制功能的M2表型以及降低吞噬能力等机制促进肺癌的发展^[46]^。总之，CCL4可以调节TME中所存在细胞的组成和关键免疫细胞的功能，在抗肿瘤免疫应答过程中起着关键作用。

**图1:**
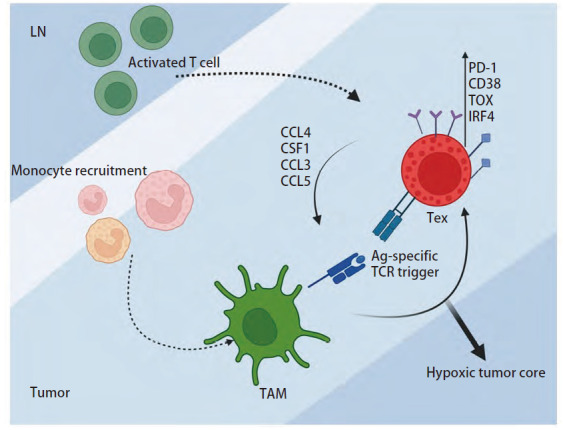
CCL4招募并诱导单核细胞分化成TAMs后与Tex形成持久突触在缺氧环境下加剧T细胞耗竭

### 2.3 CCL4单核苷酸多态性（single nucleotide polymorphisms, SNP）对肿瘤预后的影响

SNP是最为普遍的遗传变异形式，是指由单个核苷酸变异在基因组水平上所致DNA序列的多样性和多态性。遗传因素和SNP基因分型是研究肿瘤发生风险和预后的关键，在探索和预测恶性肿瘤发病及预后中有着重要的价值。已有研究^[47,48]^显示部分SNP基因会明显影响个体对肺癌患病的易感性。关于CCL4趋化因子基因中的SNP目前已有报道与肺癌、乳腺癌、口腔鳞癌、肝细胞癌的预后相关^[29,39,49,50]^。在华裔队列中的研究^[50]^发现，在肺癌队列关于CCL4基因的SNP研究中rs1634507携带GT+TT杂合子相对于GG野生型携带者发生肺癌的风险更低，与AA杂合子相比，rs10491121表现为AG+GG杂合子的人群有着更高的罹患肺癌的风险。G/A/G和T/A/A的CCL4单倍体分别明显降低和升高了罹患肺癌的风险。在肝癌队列中CCL4基因rs10491121多态性A/G杂合子的受试者与A/A基因型受试者相比，发生肝细胞癌的风险显著降低至0.665倍^[49]^。在OSCC中与AA基因型患者相比，rs10491121A/G基因型发生OSCC风险较低，而CCL4基因rs1634507的T/T纯合子与OSCC易感性关系较为明显^[39]^。此外，在乳腺导管管腔A亚型和管腔B亚型的乳腺癌患者中，与AA基因型患者相比，rs10491121的A/G基因型患者发生淋巴结转移的可能性较小^[29]^。以上这些发现都充分体现了CCL4的SNP对肿瘤预后的明显影响作用，值得我们进一步探讨。

### 2.4 CCL4在生物信息数据库中的探索

基因表达综合数据库（Gene Expression Omnibus, GEO）是国家生物技术信息中心（National Center for Biotechnology Information, NCBI）中最具代表性的基因表达数据库，是目前世界上最大的基因表达芯片数据库之一。利用数据库中关于人类肿瘤的相关重要信息数据，如蛋白质组学、基因组学、微生物和代谢组学数据，通过生信分析的方法可为预测肿瘤发生发展及治疗疗效提供可靠的依据，也是探索肿瘤诊断和治疗靶点的有效方式。目前通过生信分析发掘的重要成果^[51]^已为肿瘤临床实践提供了不菲的价值。探索CCL4在NSCLC TME中作用和特点的生信分析研究^[52]^发现：（1）在NSCLC中高表达的CCL4与免疫治疗疗效密切相关的免疫细胞受体信号通路呈明显正相关，如B细胞受体信号通路、T细胞受体信号通路、自然杀伤（natural killer, NK）细胞介导的细胞毒性通路、抗原呈递信号通路等；（2）CCL4的表达水平与NSCLC免疫治疗中最关键的效应T细胞（CD8^+^ T细胞）的浸润水平呈显著正相关（肺腺癌中CD8^+^ T细胞r=0.565，P<0.001；肺鳞癌中CD8^+^ T细胞r=0.467，P<0.001），然而CD8^+^ T细胞和B细胞浸润水平与肺腺癌患者生存呈明显正相关；（3）CCL4的表达水平与PD-L1的表达呈现出显著相关（肺腺癌中r=0.66，P<0.001；肺鳞癌中r=0.436，P<0.001）。生信分析的结果更加具体地体现出CCL4在NSCLC患者抗肿瘤免疫微环境中的关键作用和特点，也预示了在肺癌免疫治疗疗效预测中CCL4的潜在重要价值。

## 3 CCL4/CCL5/CCR5轴作为抗肿瘤治疗的靶点

CCL4/CCL5/CCR5轴信号传导在通过免疫抑制促进肿瘤生长方面具有多效性，如刺激血管生成、代谢重编辑、干细胞扩增等^[53]^。该信号通路轴可通过多种机制^[54]^促进肿瘤进展（图2），包括：（1）促肿瘤生长；（2）促进重塑细胞外基质；（3）促进TME中肿瘤细胞迁移导致转移；（4）支持癌症干细胞扩增；（5）使癌细胞耐药；（6）削弱DNA损伤药物的细胞毒性；（7）代谢重编程；（8）促进血管生成；（9）招募免疫细胞和基质细胞；（10）诱导巨噬细胞的免疫抑制极化。

**图2:**
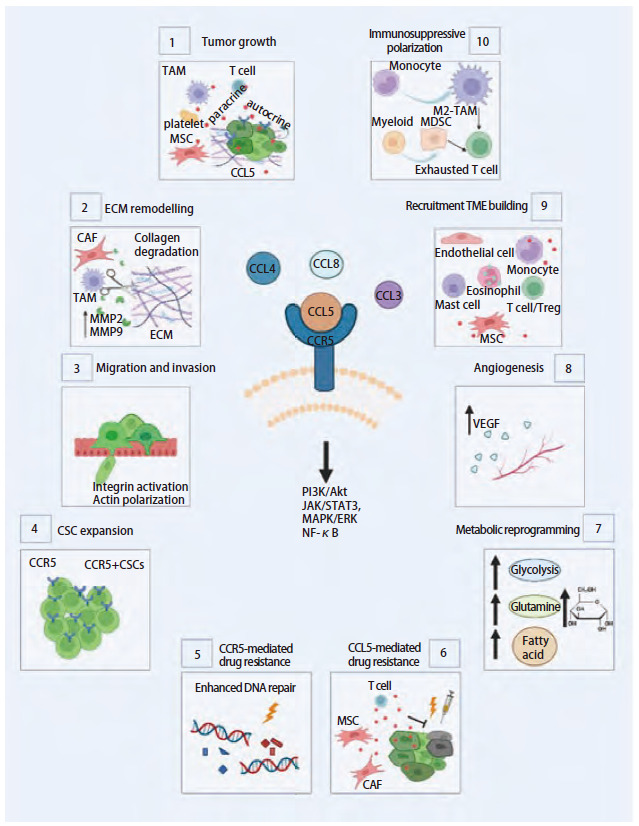
CCL4/CCL5/CCR5轴促进肿瘤进展的多种机制

基于CCR5结合趋化因子（包括CCL4、CCL3、CCL5等）在恶性肿瘤发生发展及转移等病理活动中的作用机制，CCR5拮抗剂疗法已经被提议用于抗肿瘤治疗。在CCR5缺失的人群中缺乏明显的病理抗原表型意味着接受CCR5拮抗剂治疗时不会导致严重的不良反应，也正是这一理论趋势进一步支持了这一设想的合理性和可持续性。然而CCR5拮抗剂的临床应用尚需要综合考虑CCR5结合趋化因子的实际病理生理机制去进一步探索。

依据上述CCR5信号传导通路在TME中的特征及CCL4/CCL5/CCR5轴促肿瘤进展的多种机制，目前已有针对CCL4、CCL5及CCR5的拮抗剂应用于抗肿瘤治疗的临床试验中。当下至少有4种CCR5拮抗剂正在抗肿瘤临床试验中进行评估，其中比较有代表性的CCR5拮抗剂药物有小分子CCR5拮抗剂（Maraviroc和Vicriviroc）和抗CCR5的单克隆抗体（Leronlimab和BMS-813160）^[55⇓-57]^。其中小分子CCR5拮抗剂Maraviroc单药在直肠癌的临床前模型和Maraviroc联合化疗在直肠癌临床研究（NCT01736813）中的研究^[58]^结果表明，以CCR5为靶点使用小分子CCR5拮抗剂Maraviroc可促进巨噬细胞向抗肿瘤状态的极化，研究中3/5的患者接受了Maraviroc治疗和化疗，受试者客观部分缓解的结果表明，靶向CCR5治疗对CCR5阳性且具有免疫抑制活性的髓系浸润肿瘤有明显的抗肿瘤效果。

## 4 总结和展望

随着肿瘤免疫学的发展和免疫治疗时代的来临，通过阻断免疫检查点来对抗免疫抑制的免疫疗法已经在抗肿瘤临床中获得了巨大的成就^[59,60]^，免疫治疗通过调整TME，激活并增强机体自身的效应免疫细胞来抗肿瘤，革命性地改变了既往的抗肿瘤模式，也充分体现出TME在抗肿瘤免疫中的重要作用。然而，当下以ICIs为主的免疫治疗在大部分瘤种中的疗效存在着很大的不确定性。目前除了PD-L1表达在一定程度上能够指导并预测免疫治疗疗效并已在各大诊疗指南中明确推荐之外，肿瘤突变负荷（tumor mutation burden, TMB）可能是预测免疫治疗疗效的又一标志物。但目前TMB在检测方法及阈值的选择上尚无统一的标准，无法广泛契合临床，给临床诊疗带来实质性的参考和指导。

从上文罗列的目前的临床及基础研究中不难看出CCL4在肿瘤发生发展及浸润复发转移中与免疫逃逸相关的重要调控作用。CCL4基因的SNP与多种肿瘤的患病风险及疾病预后显著相关，特定的CCL4的SNP可能成为众多肿瘤诊疗过程中颇具潜在价值的生物标志物，不同癌种相关的CCL4的SNP可用以预测肿瘤的发生、进展和转移，为肿瘤的预防和诊疗工作带来指导价值。在生物信息学分析中也看到CCL4与NSCLC甲基化、关键免疫细胞浸润水平及特殊分子表达之间的密切关系，预示着CCL4在抗肿瘤免疫应答中的关键作用，以及CCL4在免疫治疗预测疗效方面存在的重大价值，并且目前已有针对CCL4及其受体研发的新药正在参与多项多瘤种的临床研究^[55,56]^，在部分研究^[58]^结果中已经展示出良好的抗肿瘤效应。

然而，TME是一个复杂且具有高度异质性的动态系统，其中的免疫细胞与基质成分之间的相互作用导致了TME的复杂多变性。CCL4只是在TME中调控抗肿瘤免疫的趋化因子中的一种重要亚型，往往联合并协同其他趋化因子如CCL3、CCL5及其他细胞因子共同发挥作用，所以不应该脱离TME的大环境来单独研究CCL4的特点和作用。进一步探索CCL4在肿瘤中过表达的机制和原因，控制其表达是否能够有效影响肿瘤的发生发展，CCL4能否作为预测免疫治疗疗效的生物标志物等议题，将来都可能为进一步了解TME及肿瘤免疫逃逸提供新思路，从而为指导临床诊疗带来重要价值。
